# Image-Guided Embolotherapy of Arteriovenous Malformations of the Face

**DOI:** 10.1007/s00270-022-03169-0

**Published:** 2022-06-02

**Authors:** Vanessa F. Schmidt, Max Masthoff, Richard Brill, Peter B. Sporns, Michael Köhler, Victor Schulze-Zachau, Martin Takes, Denis Ehrl, Daniel Puhr-Westerheide, Wolfgang G. Kunz, Mwivano Dunstan Shemwetta, Eric M. Mbuguje, Azza A. Naif, Abizer Sarkar, Jens Ricke, Max Seidensticker, Walter A. Wohlgemuth, Moritz Wildgruber

**Affiliations:** 1 Department of Radiology, University Hospital, LMU Munich, Munich, Germany; 2 Clinic for Radiology, University Hospital Muenster, Muenster, Germany; 3 Clinic and Policlinic of Radiology, Martin-Luther University Halle-Wittenberg, Halle (Saale), Germany; 4 Department of Neuroradiology, Clinic for Radiology and Nuclear Medicine, University Hospital Basel, Basel, Switzerland; 5 Department of Hand, Plastic and Aesthetic Surgery, University Hospital, LMU Munich, Munich, Germany; 6 Department of Radiology, Muhimbili University of Health and Allied Sciences, Dar es Salaam, Tanzania

**Keywords:** AVM, Face, Embolization, Surgical resection, Interventional radiology

## Abstract

**Purpose:**

To evaluate the safety and outcome of image-guided embolotherapy of extracranial arteriovenous malformations (AVMs) primarily affecting the face.

**Materials and Methods:**

A multicenter cohort of 28 patients presenting with AVMs primarily affecting the face was retrospectively investigated. Fifty image-guided embolotherapies were performed, mostly using ethylene–vinyl alcohol copolymer-based embolic agents. Clinical and imaging findings were assessed to evaluate response during follow-up (symptom-free, partial relief of symptoms, no improvement, and progression despite embolization), lesion devascularization (total, 100%; substantial, 76–99%; partial, 51–75%; failure, < 50%; and progression), and complication rates (classified according to the CIRSE guidelines). Sub-analyses regarding clinical outcome (*n* = 24) were performed comparing patients with (*n* = 12) or without (*n* = 12) subsequent surgical resection after embolotherapy.

**Results:**

The median number of embolotherapy sessions was 2.0 (range, 1–4). Clinical outcome after a mean follow-up of 12.4 months (± 13.3; *n* = 24) revealed a therapy response in 21/24 patients (87.5%). Imaging showed total devascularization in 14/24 patients (58.3%), including the 12 patients with subsequent surgery and 2 additional patients with embolotherapy only. Substantial devascularization (76–99%) was assessed in 7/24 patients (29.2%), and partial devascularization (51–75%) in 3/24 patients (12.5%). Complications occurred during/after 12/50 procedures (24.0%), including 18.0% major complications. Patients with subsequent surgical resections were more often symptom-free at the last follow-up compared to the group having undergone embolotherapy only (*p* = 0.006).

**Conclusion:**

Image-guided embolotherapy is safe and effective for treating extracranial AVMs of the face. Subsequent surgical resections after embolization may substantially improve patients’ clinical outcome, emphasizing the need for multimodal therapeutic concepts.

**Level of Evidence:**

Level 4, Retrospective study.

**Supplementary Information:**

The online version contains supplementary material available at 10.1007/s00270-022-03169-0.

## Introduction

Within the framework of vascular malformations, arteriovenous malformations (AVMs) are probably the most challenging ones to treat [1]. Due to the high-flow shunting, hemodynamic effects may lead up to peripheral ischemia adjacent to the AVM. Patients may present with pain, bleeding, hyperemia, ulceration, and gangrene up to high-output cardiac failure [2, 3]. Extracranial vascular malformations frequently affect the head and neck area [4]. There are several reports following image-guided embolization of lesions related to this localization [5–7], but there has been rarely a distinct evaluation of high-flow malformations involving primarily the face [8]. The complex anatomy of the face is based on multiple small functional units containing dense innervation and limited soft tissue coverage. Consequently, there is a relevant risk for nerve injury, necrosis, and aesthetic disfigurement.

The purpose of this multicenter study was to evaluate the safety and clinical outcome of image-guided embolotherapies of high-flow malformations of the face, either alone or in conjunction with subsequent surgical resection and reconstruction.

### Materials and Methods

This retrospective multicenter study was approved by the local ethics committee (protocol No.: 21–0943, 10/06/2021) and was performed in accordance with the regulations of the Helsinki Declaration of 2013. All patients were recruited via Interdisciplinary Vascular Anomalies Centers at 5 tertiary care university hospitals. Data collection was performed using electronic patient records as well as the Picture Archiving and Communication System (PACS) searching for corresponding diagnosis related groups (DRGs). Arteriovenous malformations (AVMs) were diagnosed based on the combination of patient history, physical examination, and imaging using magnetic resonance imaging (MRI) and duplex ultrasound [9, 10]. Clinical and angiographic classifications were assessed according to Schobinger and Cho [11]. All malformations predominantly affected the face, patients involving the neck or head without the face were excluded. Similarly, patients who underwent diagnostic workup only without invasive treatment were excluded. The indications for interventional treatment were pain, swelling, bleeding, peripheral ischemia presenting with local dystrophy or ulceration, cosmetic disfigurement, and accompanying functional sequelae (such as epiphora). Patients presenting AVMs associated with syndromic anomalies were also included.

#### Procedural Details

All procedures were conducted under general anesthesia. The embolotherapies were performed under real-time ultrasound and fluoroscopic guidance, mostly using ethylene–vinyl alcohol copolymer (EVOH)-based liquid agents Onyx 18, 20, 34 (Medtronic, USA; numbers indicating viscosity in centipoise) and Squid 12, 18, 34 (Balt Germany GmbH, Germany; numbers indicating viscosity in centipoise). Further embolic agents include precipitating hydrophobic injectable liquid (PHIL) (Microvention Inc., USA; number indicating concentration in weight/weight) and gelified ethanol (1A Medical AG, Switzerland). The choice of different embolic agents was based on personal preference. The procedures (*n* = 50) were mostly conducted using the plug-and-push technique via the transarterial route (45/50, 90.0%) [12] including 4/50 cases (8.0%) with additionally percutaneous access. Besides this, percutaneous only as well as combined transvenous and percutaneous approach was used in 4/50 (8.0%) and 1/50 (2.0%) treatments. For percutaneous access to the nidus duplex, ultrasound guidance was routinely applied. For arterial access inguinal route was applied. Five or 6 F sheath equipment was used routinely, followed by 4/5 F guiding catheters and subsequently using microcatheters with a detachable tip (such as 1.5 and 3 cm, Apollo™, Medtronic, USA) in a triaxial manner. Patients were discharged at day 3–5 following the procedure, with low molecular weight heparin for 7 days. Depending on the extent of the lesion, clinical response to therapy, and course of clinical symptomatology, repeated embolization sessions were performed or alternatively, subsequent surgical resection of the embolized AVM tissue and, if necessary, defect reconstruction (via a myofascial flap) was performed. Surgical treatment options were discussed in an interdisciplinary setting, and procedures were conducted by plastic or maxillofacial surgery.

#### Follow-Up

The patients were seen within a standardized follow-up regime in the 5 centers involved. The first follow-up visit was performed at 1–3 months after each treatment session including repeated MRI examinations. In case of insufficient improvement of symptoms or residual perfused lesion being present, an additional embolization session was planned. In case of no additional treatment the next follow-up was scheduled at 6 months, again comprising a clinical examination as well as MRI. In case of no residual AVM left or after subsequently performed surgical resection, repeated follow-up was performed annually.

#### Outcome Evaluation After Treatment

All available pre-procedure and follow-up clinical data were analyzed with respect to demographic patient data, lesion classification, procedural characteristics, clinical response, degree of AVM devascularization, and complication rates. For clinical outcome of embolotherapies at last follow-up, a patient-reported evaluation was conducted using the following grading scale: symptom-free, partial relief of symptoms, no improvement, and clinical progression despite embolization. Objective outcome was assessed using pre-procedural MR-angiography images compared to those obtained after the last embolization ± surgical treatment. Thereby, imaging findings (percentage of AVM devascularization) were subdivided into 5 categories: total devascularization (100%), substantial remission (76–99%), partial remission (51–75%), failure (< 50%), and progress. These examinations were evaluated blinded to the clinical outcome by the consensus of one interventional radiologist (MM) with six years and one radiology resident (VFS) with four years of experience in diagnostic MRI. Peri- and post-procedural complications were classified into minor and major adverse events (AE) according to the Cardiovascular and Interventional Radiological Society of Europe (CIRSE) classification system [13].

#### Statistical Analysis

Descriptive statistics were used to analyze the distribution of patients among the different categories. Kolmogorov–Smirnov (K–S) test was used for the assessment of normality. Data are presented as means (± standard deviation) in case of normal distribution or as medians (range, minimum–maximum) for skewed distribution. Sub-analyses were performed depending on subsequent surgical resections using Pearson`s Chi-squared test for categorial data. All statistical testing was conducted using SPSS (version 26.0, IBM Corp., USA), with *p* < 0.05 considered significant.

## Results

### Patient Characteristics

A total of 28 consecutive patients, 14 males and 14 females, underwent a total of 50 image-guided embolotherapies between 2010 and 2021 (Table 1). The median age was 24 years (range, 0.5–77 years) at treatment initiation. Overall, the 28 AVMs, including 4 AVFs, presented frontal (3/28, 10.7%), orbital (3/28, 10.7%), nasal (5/28, 17.9%), temporal (5/28, 17.9%), buccal (7/28, 25.0%), labial (5/28, 17.9%), mental (6/28, 21.4%), and auricular (2/28, 7.1%) involvement. One patient (1/28, 3.6%) presented a lesion extending to 4 of these anatomical areas, 1/28 patient (3.6%) to 3 areas, and 5/28 patients (17.9%) to 2 areas. In the remaining 21/28 cases (75.0%), only 1 anatomical area of the face was involved. The cohort comprised 1/28 (3.6%) case of AVM associated with a syndromic anomaly (CM-AVM syndrome). Cho`s classification [11] showed mostly type IIIb (15/28, 53.6%) and type II (7/28, 25.0%), while 4/28 lesions (14.3%) were categorized as type I (= AVF) and 2/28 lesions (7.1%) as type IIIa. There was a mean time of 8.1 years (± 9.7 years) between the initial diagnosis and the first interventional treatment. Part of the cohort (13/28, 46.4%) had undergone previous treatment, either by partial surgical resection (4/28, 14.3%), incomplete embolization (6/28, 21.4%), or both (3/28, 10.7%).

**Table 1:** Patient characteristics of study cohort

Characteristic				Cohort (total, *n* = 28)	Cohort (follow-up, *n* = 24)
Age at diagnosis Mean (± SD)				19.4 (± 18.8)	
Men				14 (50.0%)	
Lesion size (mL) Mean (± SD)				20.8 (± 29.8)	
*Cho classification*					
Type I				4 (14.3%)	
Type II				7 (25.0%)	
Type IIIa				2 (7.1%)	
Type IIIb				15 (53.6%)	
*Schobinger classification*				*At treatment initiation*	*At terminal follow-up*
Stage I (quiescence)				2 (7.1%)	21 (87.5%)
Stage II (expansion)				16 (57.1%)	2 (8.3%)
Stage III (destruction)				10 (35.7%)	1 (4.2%)
Stage IV (decompensation)				0 (0.0%)	0 (0.0%)
*Involved anatomical areas*					
Frontal				3 (10.7%)	
Orbital				3 (10.7%)	
Nasal				5 (17.9%)	
Temporal				5 (17.9%)	
Buccal				7 (25.0%)	
Labial				5 (17.9%)	
Mental				6 (21.4%)	
Auricular				2 (7.1%)	
*Treatment rationales*					
Pain				28 (100%)	
Local bleeding				9 (32.1%)	
Peripheral ischemia				16 (57.1%)	
Local swelling				18 (64.3%)	
Cosmetic disfigurement				13 (46.4%)	
Accompanying sequelae				1 (3.6%, epiphora)	
Right heart insufficiency				0 (0.0%)	
*Symptom graduation*				*At treatment initiation*	*At terminal follow-up*
None				0 (0.0%)	12 (50.0%)
Light				2 (7.1%)	7 (29.2%)
Moderate				14 (50.0%)	3 (12.5%)
Strong				9 (32.1%)	2 (8.3%)
Very strong				3 (10.7%)	0 (0.0%)

*SD* standard deviation

## Clinical Outcome

The median number of image-guided embolotherapies per patient was 2.0 (range, 1–4). For procedural details, see Table 2. In 9/50 procedures (18.0%), there were 2 different embolic agents used. The median injected volume of the primary embolic agent was 3.0 mL (range, 0.8–4.5 mL), and of the additionally applied second agent was 2.5 mL (range, 1.5–7.5 mL) per session. The mean follow-up period after the last embolization session ± surgical treatment was 12.4 (± 13.3) months. Final clinical follow-up (after last embolization ± surgical treatment) was obtained for 24/28 patients (85.7%); thereof, the evaluation revealed an overall response of 21/24 patients (87.5%) including mainly symptom-free presentation (12/24, 50.0%) and partial relief of symptoms (9/24, 37.5%). There was no clinical improvement in 3/24 patients (12.5%) at last obtained follow-up. No patient presented with clinical progression following embolotherapy.

**Table 2:** Procedural data of study

Characteristic		Cohort (total, *n* = 28)		Primary embolic agent (*n* = 50 procedures)	Additional agent used (*n* = 9 procedures)
Age at treatment initiation, median (range)		24 (0.5–77)			
*Number of interventional procedures*					
1		11 (39.3%)			
2		10 (35.7%)			
3		5 (17.9%)			
4		1 (3.6%)			
Hospitalization in days, mean (± SD)		3.4 (± 0.8)			
Intensive care for 1 day		2 (7.1%)			
Subsequent resection (previously planned)		12 (42.9%)			
*Type of embolic agent*					
Onyx 18				26 (52.0%)	3 (33.3%)
Onyx 20				4 (8.0%)	0 (0.0%)
Onyx 34				2 (4.0%)	1 (11.1%)
Squid 12				0 (0.0%)	3 (33.3%)
Squid 18				7 (14.0%)	0 (0.0%)
Squid 34				1 (2.0%)	2 (22.2%)
PHIL 25				6 (12.0%)	0 (0.0%)
Gelified ethanol				4 (8.0%)	0 (0.0%)

*SD* standard deviation

## Imaging Outcome

Post-treatment imaging at final follow-up (*n* = 24) revealed total devascularization (100%) in 14/24 patients (58.3%) including the 12 patients with subsequential surgical resection as well as 2 additional patients after interventional treatment only. Surgical resection comprised a complete resection of the nidus in all cases with however incomplete resection of all embolized feeding and draining vessels, as detected on post-surgery imaging studies. In three cases, a temporal myofascial flap was required for defect reconstruction. Substantial devascularization (76–99%) was assessed in 7/24 patients (29.2%), and partial devascularization (51–75%) in 3/24 patients (12.5%). In total, this resulted in an overall objective response of 100%.

## Safety and Complications

**Fig. 1:**
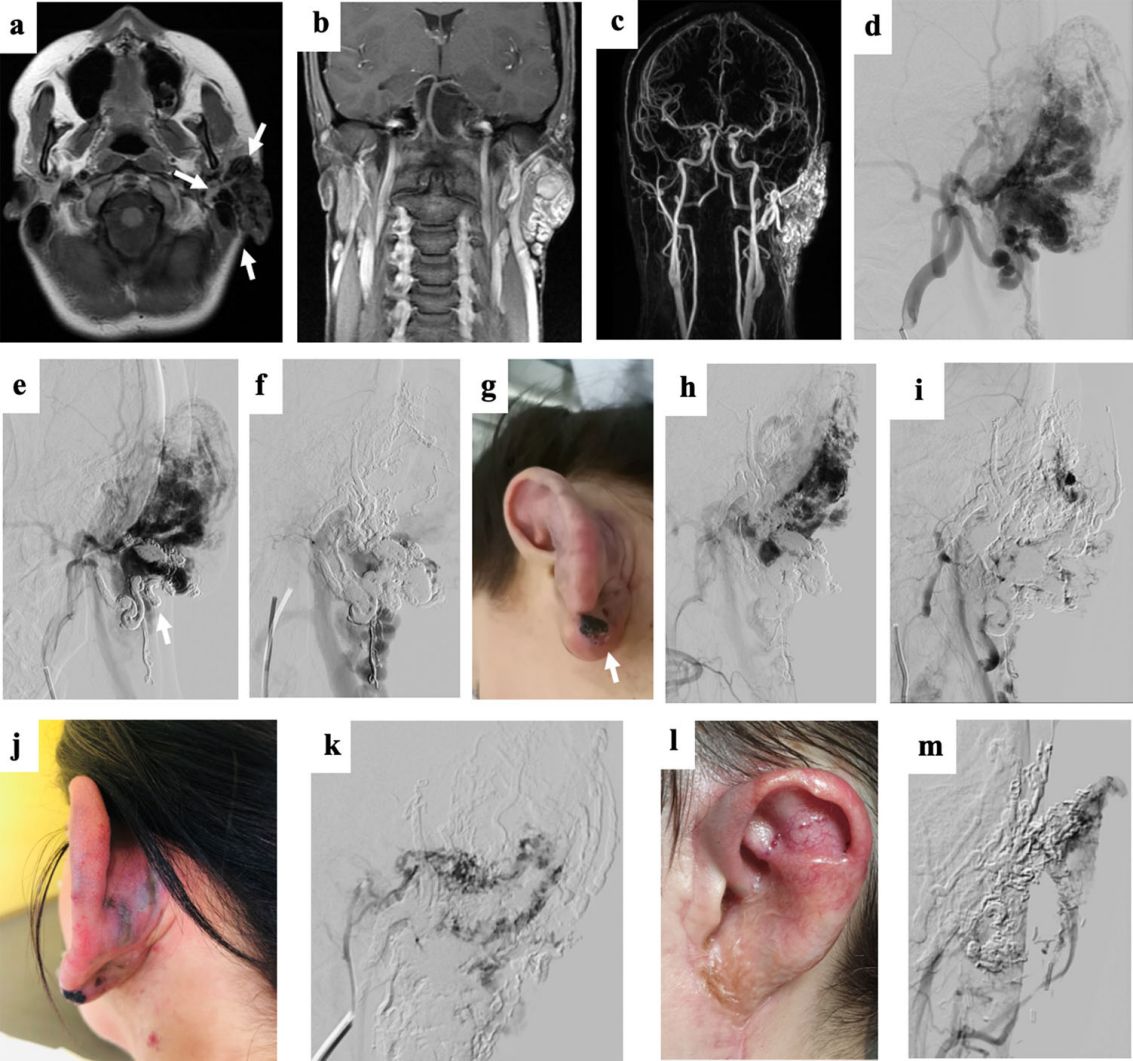
30-year old female patient presenting with an extensive arteriovenous malformation (AVM) left (peri)auricular. After 4 sessions of embolotherapies with corresponding near-total devascularization of the malformation, previously planned two-step microsurgical resection and defect reconstruction using a free fascia flap was performed. **(a–c)** Preprocedural axial T1-weighted (T1-w) MR image, coronar T1-w MR image, and coronar MR-angiography image present the extent of the left (peri)auricular AVM with ubiquitous involvement of the auricle (arrows) and at least 4 arterial feeders (2 × from facial artery, 2 × from posterior auricular artery, 1 × from occipital artery) as well as venous drainage into the external jugular vein. **(d–e)** Periprocedural digital subtraction angiography (DSA) images during 1st embolotherapy show flow characteristics of the lesion and the successfully embolized vascular structures at the caudal part of the earlobe (*arrow*). **(f)** Periprocedural DSA image during 2nd embolotherapy shows the newly embolized vascularized components of the malformation. **(g)** Clinical photograph 1 day before the 3rd embolotherapy presents the enlarged and prominent ear auricle and lobule, transparent vascular structures of the malformation, and a small, long-term necrotic area on the caudal ear lobe. **(h + i)** Periprocedural DSA images during 3rd embolotherapy demonstrate remaining vascularized components of the malformation (*arrow*) as well as successfully embolized cranial components of the lesion. The patient presented with visual disorders directly post-procedural and MRI revealed 2 subtle restricted-diffusion lesions including the visual cortex, most likely due to periprocedural small air emboli. These findings were entirely regressive while preventive monitoring at the stroke unit. **(j)** Clinical photograph 3 days after the 3rd embolotherapy. **(k)** Periprocedural DSA image during the 4th embolotherapy shows arterial bleeding occurred after minor manipulation under anesthesia, due to previously unnoticed secondary abscessing after the 3rd session. After abscess draining and embolization of the arterial bleeding using histoacryl/lipiodol mixture, Onyx embolization of the remaining AVM components was successfully performed in the same session. **(l)** Clinical photograph after initially planned surgical resection of the devascularized AVM tissue including parts of the ear auricle and lobe and defect reconstruction with means of a free serratus fascia flap both successfully performed 3 months after the 4th session. **(m)** Diagnostic DSA image at final follow-up (21 months after the last treatment) presents a good perfusion of the apical ear auricle as well as no novel AVM components. Clinically, the patient appeared without any symptoms or further signs of AVM recurrence

Peri- and post-procedural complications were observed following 12/50 procedures (24.0%, CIRSE grade 1–4). For detailed description of the adverse events, see Supplementary Material. In total, the major complication rate (> CIRSE grade 2) was 9/50 (18.0%), see Fig. 1.

## Impact of Subsequent Surgical Resection

There were significant differences of the clinical outcome at the last follow-up (*n* = 24) depending on the treatment concept (embolotherapy vs. embolotherapy + subsequent surgery; *p* = 0.006): after subsequent surgical resection of the embolized nidus (*n* = 12), there was mainly symptom-free presentation (10/12, 83.3%) whereas the group without subsequent surgery (*n* = 12) mostly presented with partial relief of symptoms (7/12, 58.3%). No improvement of symptoms at final follow-up was only noted in the group without subsequent surgery (3/12, 25.0%) (Table 3).

**Table 3:** Clinical outcome at final follow-up (*n* = 24) depending on the treatment concept

Characteristic	Embolotherapy only (*n* = 12)	Embolotherapy + subsequent surgery (*n* = 12)
Symptom-free	2 (16.7%)	10 (83.3%)
Partial relief of symptoms	7 (58.3%)	2 (16.7%)
No improvement of symptoms	3 (25.0%)	0 (0.0%)
Progression despite embolization	0 (0.0%)	0 (0.0%)

*SD* standard deviation

### Discussion

In this study evaluating image-guided embolotherapy of AVMs primarily affecting the face, a high overall clinical and objective response rate (88% and 100%, respectively) is being reported, accompanied by a moderate rate of 18% major complications. The group with subsequent additional surgical resections presented more frequently symptom-free compared to embolization only.

To reduce the risk of recurrence, which is increased after incomplete therapy, embolization and surgery aim at the entire occlusion or removal of the nidus, and, at best, of its feeding and draining vessels [14]. Several studies reported on AVMs of head and neck but were rarely restricted to the face; thus, comparison of our data with the literature is limited [6, 15, 16].

Complete devascularization was obtained in the most cases (58%) including the 12 patients with subsequential surgical resection. These results are similar to previously published studies of embolotherapy of extracranial AVMs [6, 17, 18], though there has to be noted that in our cohort a higher percentage received subsequent surgical resection (41%).

While using EVOH-based liquid agents in most procedures we found an overall and major complication rate of 24% and 18%, respectively. The treatment safety in the present study is comparable to the reported literature on embolization of AVMs using EVOH [12, 17, 19]. The majority of complications were post-procedural progressive necroses, in 2 cases accompanied by additional circumscribed hypoesthesia and in 1 case by a secondary infection in the further course. Skin necrosis and nerve injury are the most frequently reported adverse events after embolotherapy of AVMs in particular when using ethanol, a rather aggressive embolic agent [16]. Ethanol has been described as safe and effective embolic agent, even for embolization in the head and neck region [20]. The advantage of ethanol is that essentially endothelial ablation is achieved, which might reduce the risk of recurrence. However, as severe complications including death have been reported with the use of pure ethanol [21], EVOH-based embolic agents are becoming more common. Important when using EVOH is that the embolic is pushed into the nidus of the AVM, proximal occlusion of feeding or draining vessels only needs to be avoided as persistent perfusion of the nidus can lead to recurrence and growth. Besides the plug-and-push techniques used in our cohort, other approaches such as the pressure cooker technique or occlusion microballoons can be applied [22]. When performing EVOH embolization with subsequent surgery, the surgeon needs to be aware of possible interference of EVOH with cauterization devices. Additionally, in case of EVOH-based liquid embolization material mixed with Tantalum, one has to be aware that in superficial AVM lesions the application can result in tattooing of the skin, which can be avoided when using EVOH-based agents mixed with iodine or glue instead for superficial lesions with excellent results [20].

Additionally, it should also be noted that the classification of complications according to CIRSE used here for AVMs affecting the face may not specifically reflect important relevant consequences related to this specific localization, in particular aesthetic disfigurement or nerve damage, which significantly affects patients in the long term.

In our cohort, 12 patients underwent subsequent surgical resection of the residual lesions following the initial treatment plan. At these, due to the clinical presentation at baseline, a combined approach was considered the most appropriate. The comparison of the clinical outcome at the last follow-up depending on the treatment concept (± subsequent surgery) revealed a higher percentage of patients presenting symptom-free in the cohort with subsequent surgical resections. These results highlight that in AVMs complete occlusion with surgical removal of the nidus should be the treatment goal, if achievable and reasonable feasible [23]. Subsequent surgery of embolized lesions may not only result in reduced recurrence rates [24], but we could demonstrate significant improvement in patient’s clinical outcome. As resections were often performed immediately after the last embolization without MRI in between, it is retrospectively difficult to determine whether these patients achieved a good outcome due to the additional resection or due to complete devascularization achieved in previous embolizations.

This multicenter analysis has several limitations. First, it represents a retrospective design including a consecutive lack of standardized follow-up data available for the total cohort. Second, standardized disease-related questionnaires to evaluate standardized the specific symptomatology and functional impairments were not used as a tool for the clinical response. Third, the methodological approach to measure lesion devascularization in form of a categorization in percentages is not validated; nevertheless, it is the most frequently used method reported in the literature [25]. New imaging modalities assessing functional parameters of vascular malformations may improve therapy monitoring and response evaluation in the future [26]. These aspects highlight the general complexity in studying this orphan disease, as there are presently no established criteria for the assessment of the clinical and imaging response. Fourth, it should be noted that our cohort was treated with a variety of embolic agents lowering the consistency and comparability of the procedural data, even if in the majority EVOH-based liquid agents had been used. Additionally, in regard of the high recurrence rate of high-flow vascular malformations commonly manifesting after a longer term, the mean follow-up time of about 12 months after the last embolotherapy ± surgical treatment was relatively short. This also means that it was not yet practical to determine a recurrence rate, which is considered an important outcome parameter. Moreover, conclusions regarding the long-term efficacy of the proposed approach were not feasible.

However, the results of this study emphasize the use of endovascular embolotherapy and subsequent surgical resection also for the therapy of AVMs of the face. Image-guided embolization is both effective and safe. Subsequent surgical resection after sufficient devascularization may substantially improve patient’s clinical outcome highlighting the need for interdisciplinary treatment concepts.

## Supplementary Information

Below is the link to the electronic supplementary material.

Supplementary file1 (DOCX 13 kb)
