# Supplementary material for: Image-Guided Embolotherapy of Arteriovenous Malformations of the Face
Source: Cardiovasc Intervent Radiol. 2022 Jun 2;45(7):992–1000. doi: 10.1007/s00270-022-03169-0 (PMC9226106; doi:10.1007/s00270-022-03169-0)
Supplement: Supplementary file 1 — Supplementary file1 (DOCX 13 kb) [file 270_2022_3169_MOESM1_ESM.docx]

Supplementary Information

*Detailed description of complications*

Hereof, non-target embolization was noted during 2/50 embolotherapies (4.0%, CIRSE grade 1) with consequent persistent skin darkening (tattoo effect) in both cases. After 7/50 procedures treatment-related new skin necrosis occurred (14.0%, CIRSE grade 3). In 4 patients, the necrotic areas were conservatively resolved following intensified wound care during a prolonged hospitalization, but without any sequelae. In 2 of the 7 cases the progressive necroses were accompanied by postprocedural temporary hypoesthesia and swelling at the affected site, totally reversible as a result of intensified wound care. In 1 of the 7 cases the progressive necrosis was followed by a secondary late infection, requiring anti-infective treatment and reconstruction by plastic surgery. This patient had not received a subsequent surgical resection of the residual AVM tissue but presented with partial relief of symptoms at last follow-up. After 1/50 procedures (2.0%, CIRSE grade 4) treatment-related hypoesthesia of the buccal facial surface occurred, which was partially reversible. Another patient suffering from an extensive AVM (peri)auricular presented with visual disorders directly after the third embolotherapy. Immediate CT-angiography showed no abnormal findings while MRI revealed 2 subtle occipital restricted-diffusion lesions in the visual cortex which were attributed to periprocedural small air emboli (1/50 procedures, 2.0%, CIRSE grade 2). The visual impairment resolved completely during appropriate care on the stroke unit. In the same patient, arterial bleeding and pyorrhea occurred following the 4th embolotherapy (1/50 procedures, 2.0%, CIRSE grade 3). Abscess drainage was performed and the bleeding as well as the remaining AVM parts were successfully embolized in the same session. During hospitalization the patient was treated with intravenous antibiotics. Three months hereafter, the initially planned surgical resection of the devascularized AVM tissue including parts of the ear auricle and lobe was performed in a two-step procedure. Defect reconstruction was conducted using a free serratus fascia flap while retaining the patient's genuine cartilage. At last follow-up (21 months after the last treatment), the patient presented symptom-free and without any signs of AVM recurrence.
